# Anti-Inflammatory Effects of C1q/Tumor Necrosis Factor-Related Protein 3 (CTRP3) in Endothelial Cells

**DOI:** 10.3390/cells10082146

**Published:** 2021-08-20

**Authors:** Andreas Schmid, Ann-Kathrin Vlacil, Jutta Schuett, Thomas Karrasch, Bernhard Schieffer, Andreas Schäffler, Karsten Grote

**Affiliations:** 1 Department of Internal Medicine III, University of Giessen, 35390 Giessen, Germany; thomas.karrasch@innere.med.uni-giessen.de (T.K.); andreas.schaeffler@innere.med.uni-giessen.de (A.S.); 2 Department of Cardiology and Angiology, Philipps-University Marburg, 35037 Marburg, Germany; kochannk90@gmail.com (A.-K.V.); j.lamle@gmx.de (J.S.); Bernhard.Schieffer@uk-gm.de (B.S.); grotek@staff.uni-marburg.de (K.G.)

**Keywords:** endothelial cells, infection, inflammation, endotoxin, CTRP3, adipocytes, adipose tissue

## Abstract

The C1q/TNF-related protein 3 (CTRP3) represents a pleiotropic adipokine reciprocally associated with obesity and type 2 diabetes mellitus and exhibits anti-inflammatory properties in relation to lipopolysaccharides (LPS)-mediated effects in adipocytes, as well as monocytes/macrophages. Here, we focused on the influence of CTRP3 on LPS-mediated effects in endothelial cells in order to expand the understanding of a possible anti-inflammatory function of CTRP3 in a setting of endotoxemia. An organ- and tissue-specific expression analysis by real-time PCR revealed a considerable Ctrp3 expression in various adipose tissue compartments; however, higher levels were detected in the aorta and in abundantly perfused tissues (bone marrow and the thyroid gland). We observed a robust Ctrp3 expression in primary endothelial cells and a transient upregulation in murine endothelial (MyEND) cells by LPS (50 ng/mL). In MyEND cells, CTRP3 inhibited the LPS-induced expression of interleukin *(Il)-6* and the tumor necrosis factor *(Tnf)-α*, and suppressed the LPS-dependent expression of the major endothelial adhesion molecules *Vcam-1* and *Icam-1*. The LPS-induced adhesion of monocytic cells to an endothelial monolayer was antagonized by CTRP3. In C57BL/6J mice with an LPS-induced systemic inflammation, exogenous CTRP3 did not affect circulating levels of TNF-α, ICAM-1, and VCAM-1. In conclusion, we characterized CTRP3 beyond its function as an adipokine in a setting of vascular inflammation. CTRP3 inhibited LPS-induced endothelial expression of adhesion molecules and monocyte cell adhesion, indicating an important vascular anti-inflammatory role for CTRP3 in endotoxemia.

## 1. Introduction

Cardiovascular diseases represent a severe and worldwide health issue and are among the most common causes of premature death [1]. A significantly elevated risk of cardiovascular morbidity and mortality is associated with the continuously increasing prevalence of the metabolic syndrome [2] which is mainly driven by the global increase in obesity [3]. Vascular endothelial dysfunction, representing a key element of many cardiovascular diseases, is often associated with and aggravated by metabolic dysregulation and adipose tissue inflammation, commonly referred to as metaflammation [4], in obesity [5].

Besides its classical physiological functions in energy storage and lipid metabolism, adipose tissue represents an important endocrine organ [6,7] and its role in immune-regulatory processes linking metabolism and inflammation/immunity has been increasingly recognized in recent years [8,9]. Among the ever-growing number of identified adipokines [10], representing secretory proteins which originate from adipocytes and exert paracrine and endocrine functions, C1q/TNF-related protein 3 (CTRP3) is an important and pleiotropic factor involved in both immunological and metabolic processes. Similar to its paralog adiponectin, systemic CTRP3 is negatively associated with obesity [11,12]. Among diverse regulatory effects, CTRP3 has recently been reported to affect adipose tissue mass and obesity [13,14,15]; to exert protective effects on metabolic dysregulations, such as hepatic steatosis and insulin resistance [16,17]; and to exhibit anti-inflammatory properties in diverse pathophysiological contexts [18,19]. Of particular interest, CTRP3 antagonizes adipose tissue inflammation induced by lipopolysaccharides (LPS) through the Toll-like receptor (TLR) 4 in vitro and in vivo [20,21] and represents an effective inhibitor of metabolic inflammation mediated by free fatty acids and TLR4 [22]. On a cellular level, CTRP3 inhibits the LPS-TLR4-mediated release of the chemoattractant protein C-C motif chemokine ligand 2 (CCL2) from adipocytes [20], and of the pro-inflammatory cytokines interleukin (IL) 6 and tumor necrosis factor (TNF)-α from monocytes [23], indicating a key role of this adipokine in the paracrine regulation of adipocyte–monocyte/macrophage crosstalk both in metaflammation [4] and bacterial infection.

The endothelium, as a barrier between blood and the underlying tissue, plays an important role not only in the development of cardiovascular diseases, but also in the defense against infections. In an infectious scenario, the vascular endothelium is massively attacked by pathogens and their components are actively involved in the subsequent immune response by expressing and releasing inflammatory cytokines, controlling coagulation as well as leucocyte attraction and trafficking [24]. To date, information about a possible role of CTRP3 in endothelial processes is lacking. Based on previous reports [20,21], we hypothesized significant anti-inflammatory and protective effects of CTRP3 in LPS-induced endothelial inflammation.

## 2. Materials and Methods

### 2.1. Recombinant CTRP3

CTRP3 was expressed using the baculovirus expression system in insect H5 cells [25]. Details are described in Appendix A.

### 2.2. Mice and Cells

#### 2.2.1. LPS-Induced Systemic Inflammatory Response Syndrome (SIRS) Model

Overnight-fasted littermate male C57BL/6J mice (age 10–12 weeks) (from Charles River, Sulzfeld, Germany) were treated with intraperitoneal (i.p.) injection of either recombinant CTRP3 (dissolved in sterile PBS; dose: 10 µg per animal) or PBS (as a control). Following a pre-incubation time of 30 min, moderate inflammation was induced by i.p. injection of lipopolysaccharide (LPS; 1 µg per animal dissolved in sterile H_2_O) (control: H_2_O). Mice were euthanized 2 h after LPS injection and blood serum was sampled. These experiments were approved by the governmental Animal Ethics Committee (*Regierungspräsidium Oberpfalz*, No. 54-2532.1-14/10) and were conform to the guidelines from the directive 2010/63/EU of the European Parliament.

#### 2.2.2. Murine Endothelial Cells

Primary murine endothelial cells were isolated from lungs of male C57BL/6N wild-type mice as described previously [26]. Briefly, for each cell isolation, three mice at the age of 6–10 weeks were sacrificed by cervical dislocation. The lobes of the lung were removed under a sterile laminar flow hood and minced. Afterwards, minced lung tissue was digested using pre-warmed collagenase (2 mg/mL, Worthington Biochemical Corporation, Lakewood, NJ, USA). After triturating and straining, endothelial cells were purified using a two-step magnetic bead separation approach. Cells were incubated in Dulbecco’s Modified Eagle Medium (DMEM, Gibco, Darmstadt, Germany)/F-12, 20% fetal calf serum (FCS, PAN-Biotech, Aidenbach, Germany), 1% penicillin/streptomycin (P/S, 100 U/mL and 100 mg/mL, Sigma-Aldrich, Seelze, Germany) and endothelial cell growth supplement ECGS/H (Promocell, Heidelberg, Germany) and were used for further experiments when reaching confluence.

The murine MyEnd (myocardial endothelial) cell line was grown in DMEM with 10% FCS and 1% P/S. Endothelial properties of MyEnd cells were recently confirmed by our group [27]. The murine monocyte/macrophage cell line J774A.1 was grown in DMEM-Glutamax (Gibco) with 10% FCS and 1% P/S. Primary human endothelial cells (human umbilical vein endothelial cells, HUVECs) were obtained from Lonza (Cologne, Germany). Cells were cultured in endothelial cell growth medium (EGM-2; Lonza) that was supplemented with 2% FCS, growth factors (epidermal growth factor, vascular endothelial growth factor, fibroblast growth factor, insulin like growth factor-1) and 1% P/S.

### 2.3. Isolation of Tissues and Organs

**Figure 1:**
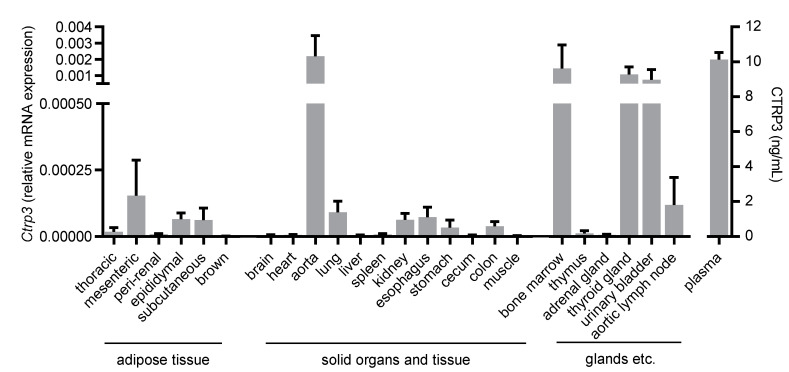
Tissue expression of Ctrp3. Ctrp3 (C1q/TNF-related protein 3) mRNA levels in different murine tissues and organs were determined by Real-time PCR and CTRP3 serum level by ELISA. *n* = 4–6 mice.

Tissues and organs were isolated from male C57BL/6N wild-type mice at the age of ~10 weeks and were disrupted using stainless steel beads (5 mm, Qiagen, Hilden, Germany) for subsequent RNA isolation and real-time PCR. Isolated tissues and organs are listed in Figure 1.

### 2.4. Real-Time PCR

For the analysis of mRNA expression, total RNA from MyEnd cells and murine tissues and organs was isolated using RNA-Solv^®^ Reagent (Omega Bio-tek, Norcross, GA, USA) following the manufacturer’s instructions and was reverse-transcribed with SuperScript reverse transcriptase, oligo(dT) primers (Thermo Fisher Scientific, Waltham, MA, USA), and deoxynucleoside triphosphates (Promega, Mannheim, Germany). Real-time PCR was performed in duplicates in a total volume of 20 µL using Power SYBR green PCR master mixture (Thermo Fisher Scientific) on a Step One Plus real-time PCR system (Applied Biosystems, Foster City, CA, USA) in 96-well PCR plates (Applied Biosystems). SYBR Green fluorescence emissions were quantified after each cycle. For normalization, expression of glyceraldehyde-3-phosphate dehydrogenase (*Gapdh*) as a housekeeping gene was determined in duplicates. Relative gene expression was calculated applying the 2^−ΔΔCt^ method. PCR primers were obtained from Microsynth AG (Balgach, Switzerland) and sequences are available upon request. Real-time PCR was performed in technical duplicates.

### 2.5. Enzyme-Linked Immunosorbent Assay (ELISA)

Supernatant from MyEnd cells and murine blood serum was analyzed for TNF-α, sVCAM-1, and sICAM-1 applying mouse-specific ELISA kits from R&D Systems (Minneapolis, MN, USA) and CTRP3 was quantified in murine blood serum applying a mouse-specific ELISA kit (LSBio, Seattle, WA, USA). ELISA was performed in technical duplicates. Prior to ELISA, serum samples were diluted in sample buffer to an appropriate range of concentrations (31.3–2000 pg/mL for CTRP3 and TNF-α, 125–8000 pg/mL for sVCAM-1 and sICAM-1). Protein concentrations were calculated from optical density applying a regression standard curve and coefficient of variation (CV) was calculated for each duplicate. Measurement was repeated for duplicates with a CV exceeding 20%.

### 2.6. Cell Adhesion Assay

MyEnd cells were plated in fibronectin-coated wells of a 24-well plate (TPP) in DMEM with 10% FCS and 1% P/S and were grown to complete confluence. Cells were cultured in DMEM with 1% FCS and were incubated with LPS (50 ng/mL, Sigma-Aldrich) or CTRP3 (10 µg/mL) for 16 h. For co-incubation conditions, CTRP3 was added to the cells 30 min prior to LPS. In parallel, J774A.1 cells were labeled with 1 μM of calcein-AM (Invitrogen) according to the manufacturer’s instructions. A total of 0.2 × 10^6^ labelled J774A.1 cells in 1 mL DMEM with 1% FCS were added to the MyEnd cells per well and co-cultured for 1 h in a 5% CO_2_ atmosphere at 37 °C. Subsequently, each well was washed three times with each 1 mL PBS. Ten high powerfield (HPF) digital images were taken before and after washing using Axio Vert.A1 microscope equipped with an AxioCam MRm camera (Carl Zeiss). Adhered calcein-AM-labeled J774A.1 cells per HPF were counted using ImageJ software (1.49v, National Institutes of Health, Bethesda, MD, USA).

### 2.7. Statistical Analysis

All data were represented as means ± SEM. Homogeneity of variance was checked by Brown–Forsythe test and data were subsequently compared using 1-way ANOVA followed by Tukey multiple comparison test (GraphPad Prism, version 6.05; GraphPad Software, La Jolla, CA). A value of *P* < 0.05 was considered statistically significant. Numbers of independent experiments were indicated in the respective figure legends.

## 3. Results

### 3.1. Ctrp3 Is Expressed in a Wide Variety of Tissues and Organs and in Endothelial Cells

**Figure 2:**
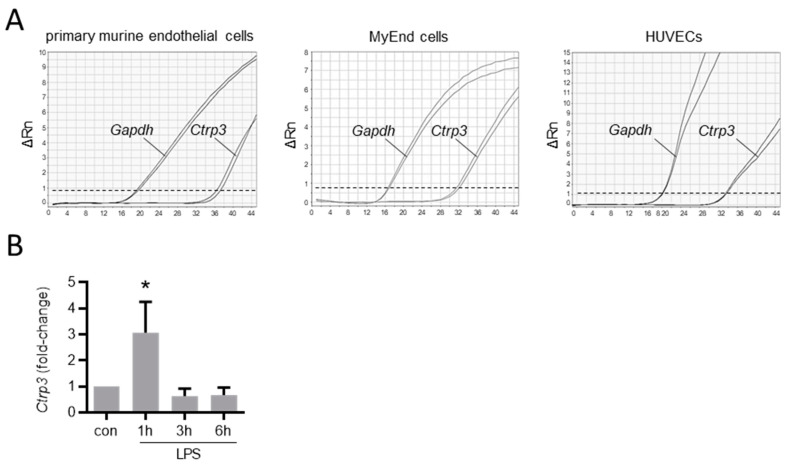
*Ctrp3* expression in endothelial cells. (**A**) The *Ctrp3* (C1q/TNF-related protein 3) mRNA expression in primary murine endothelial cells, in murine MyEnd (myocardial endothelial) cells, and HUVECs (human umbilical vein endothelial cells) was analyzed by real-time PCR. Gene expression of *Gapdh* (glyceraldehyde-3-phosphate dehydrogenase) is shown as a housekeeping gene control. Representative pictures are shown. Dashed line indicates threshold line. Rn = normalized reporter. (**B**) MyEnd cells were stimulated with LPS (lipopolysaccharides, 50 ng/mL) for the indicated period and *Ctrp3* mRNA levels were analyzed by real-time PCR. * *p* < 0.05 vs. con = unstimulated control, *n* = 5–9.

To gain an overview of the general expression of *Ctrp3* in the body, we isolated various tissues and organs from wild-type mice for subsequent real-time PCR analyses. As expected, *Ctrp3* expression was detected in adipose tissue: the highest level in mesenteric; followed by epididymal and subcutaneous tissue; and a rather low level expressed in thoracic, perirenal, and brown adipose tissue. However, *Ctrp3* was expressed at a similar level in solid organs and tissues such as the lungs, kidneys and colon and even stronger in the aorta, bone marrow, thyroid gland, and urinary bladder (Figure 1). Moreover, considerable CTRP3 levels of about 10 ng/mL in murine serum samples were determined by ELISA (Figure 1). Many of the investigated organs are well-perfused with plenty of vessels and capillaries containing endothelial cells, which have important functions, for example, in terms of infection at the interface between blood and the underlying tissue. Real-time PCR revealed well detectable *Ctrp3* levels in primary murine endothelial cells and in murine endothelial MyEnd cells, which were used for all subsequent experiments (Figure 2A). Likewise, human endothelial cells (human umbilical vein endothelial cells, HUVECs) were found to express *Ctrp3* at similar levels (Figure 2A). Stimulation of MyEnd cells with LPS resulted in a transient upregulation of *Ctrp3* (Figure 2B).

### 3.2. LPS-Induced Cytokine Expression in Endothelial Cells Is Inhibited by CTRP3

**Figure 3:**
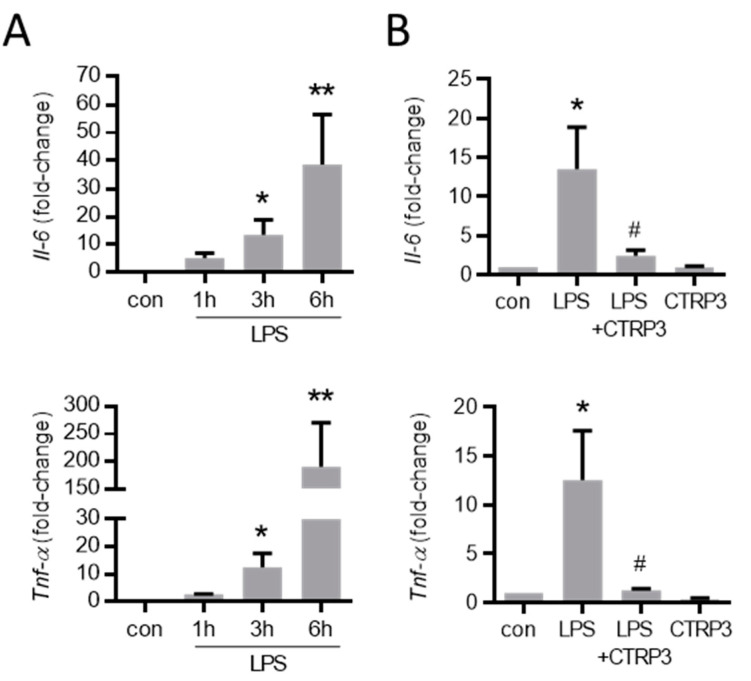
LPS-induced cytokine expression in endothelial cells is inhibited by CTRP3. (**A**) MyEnd (myocardial endothelial) cells were stimulated with LPS (lipopolysaccharides, 50 ng/mL) for the indicated period. The mRNA levels of *Il-6* (interleukin-6) and *Tnf-α* (tumor necrosis factor-α) were analyzed by real-time PCR. * *p* < 0.05, ** *p* < 0.01 vs. con = unstimulated control, *n* = 5–6. (**B**) MyEnd cells were stimulated with LPS (50 ng/mL) alone or pretreated with CTRP3 (C1q/TNF-related protein 3, 10 µg/mL, for 30 min) and CTRP3 alone for 3 h. *Il-6* and *Tnf-α* mRNA levels were analyzed by real-time PCR. * *p* < 0.05 vs. con = unstimulated control, ^#^ *p* < 0.05 vs. LPS, *n* = 4–6.

**Figure A1:**
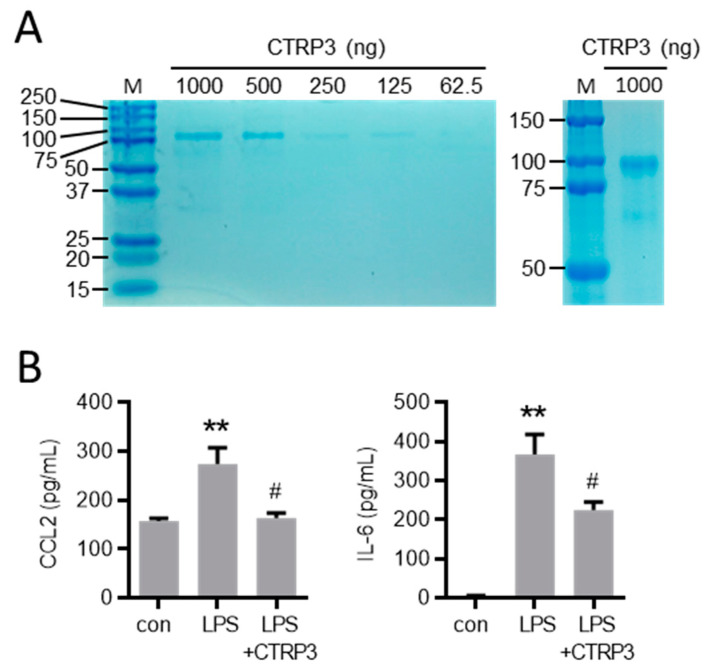
Characterization of recombinant CTRP3. (**A**) Indicated quantities of recombinant CTRP3 (C1q/TNF-related protein 3) were run on SDS-PAGE (left) and 1000 ng CTRP3 for a longer gel run (right). Gels were subsequently stained with Coomassie. M = protein size marker. (**B**) THP-1 monocyte-like cells were stimulated with LPS (10 ng/mL) alone or pretreated with CTRP3 (10 µg/mL, for 30 min) and with CTRP3 alone for 18 h. CCL2 (C-C motif chemokine ligand 2) and IL-6 (interleukin-6) protein levels in the cell supernatant were analyzed by ELISA ** *p* < 0.01 vs. con = unstimulated control, ^#^ *p* < 0.05 vs. LPS, *n* = 5–6.

As expected, LPS (50 ng/mL) strongly increased the expression of inflammatory cytokines such as interleukin-6 (*Il-6*) and the tumor necrosis factor-α (*Tnf-α*) in MyEnd cells 3 h after stimulation (Figure 3A). To investigate the effects of exogenous CTRP3 in this context, we expressed recombinant CTRP3 using the baculovirus expression system in insect cells as described earlier [25]. Recombinant CTRP3, running as a trimeric molecule at <100 kDa on SDS-polyacrylamide gels (Figure A1A), inhibited LPS-induced CCL2 and the IL-6 release from THP-1 cells in activity assays (Figure A1B). In MyEnd cells, pre-incubation with CTRP3 (30 min) significantly inhibited the LPS-induced expression of *Il-6* and *Tnf-α* after 3 h, whereas CTRP3 alone had no effect on cytokine expression (Figure 3B).

### 3.3. LPS-Induced Endothelial Adhesion Molecule Expression and Adhesion of Monocytic Cells Is Inhibited by CTRP3

**Figure 4:**
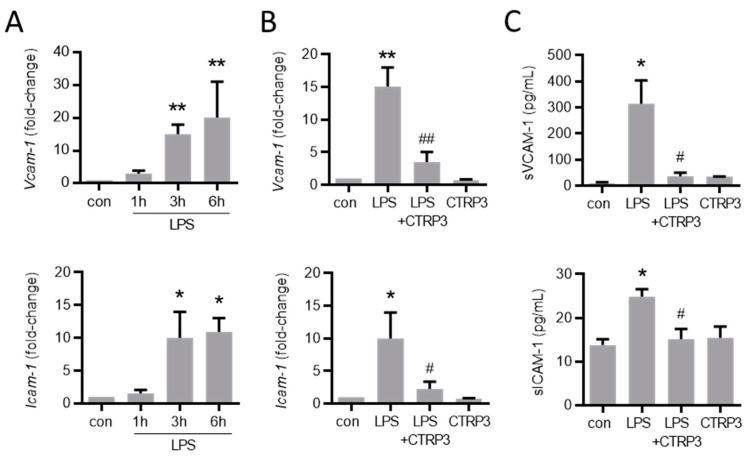
LPS-induced endothelial adhesion molecule expression is inhibited by CTRP3. (**A**) MyEnd (myocardial endothelial) cells were stimulated with LPS (lipopolysaccharides, 50 ng/mL) for the indicated period. *Vcam-1* (vascular cell adhesion molecule-1) and *Icam-1* (intercellular adhesion molecule-1) mRNA levels were analyzed by real-time PCR. * *p* < 0.05, ** *p* < 0.01 vs. con = unstimulated control, *n* = 5–6. (**B**) MyEnd cells were stimulated with LPS (50 ng/mL) alone or pretreated with CTRP3 (C1q/TNF-related protein 3, 10 µg/mL, pre-incubation for 30 min) and with CTRP3 alone for 3 h; *Vcam-1* and *Icam-1* mRNA levels were analyzed by real-time PCR. * *p* < 0.05, ** *p* < 0.01 vs. con = unstimulated control, ^#^ *p* < 0.05, ^##^ *p* < 0.01 vs. LPS, *n* = 4–6. (**C**) MyEnd cells were stimulated with LPS (50 ng/mL) alone or pretreated with CTRP3 (10 µg/mL, for 30 min) and with CTRP3 alone for 3 h. The sVCAM-1 (soluble vascular cell adhesion molecule-1) and sICAM-1 (soluble intercellular adhesion molecule-1) protein levels in the cell supernatant were analyzed by ELISA. * *p* < 0.05 vs. con = unstimulated control, ^#^ *p* < 0.05 vs. LPS, *n* = 3–5.

**Figure 5:**
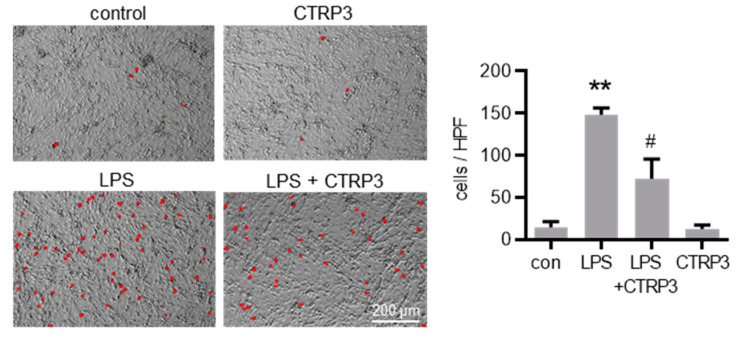
CTRP3 inhibits LPS-induced adhesion of monocytic cells on endothelial cells. Fluorescence images depicting calcein-AM-labeled J774A.1 cells on a MyEnd (myocardial endothelial) cell monolayer stimulated with LPS (lipopolysaccharides, 50 ng/mL) alone or pretreated with CTRP3 (C1q/TNF-related protein 3, 10 µg/mL, for 30 min) and with CTRP3 alone for 16 h with an additional adhesion time of 1 h and a final washing. Representative pictures are shown. HPF = high power field, Scale bar = 200 µm, ** *p* < 0.01 vs. con = unstimulated control, ^#^ *p* < 0.05 vs. LPS, *n* = 3.

**Figure A2:**
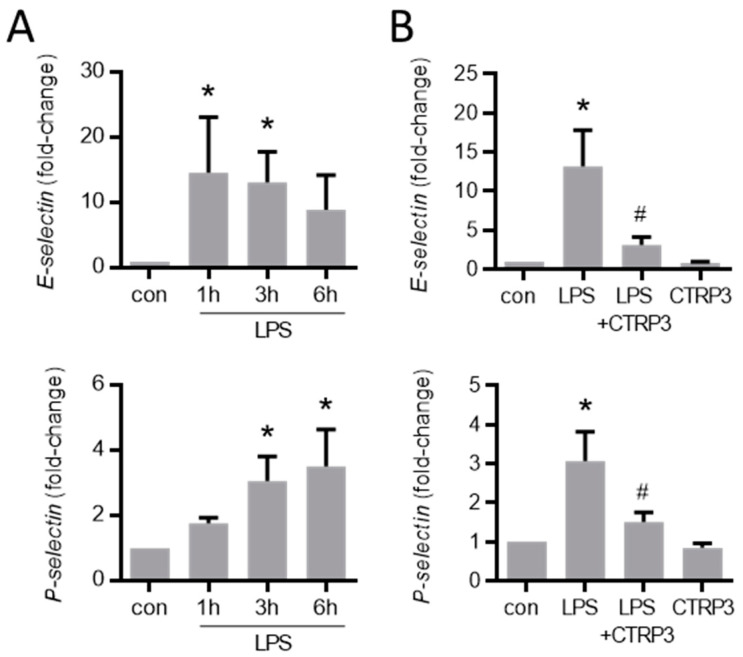
LPS-induced endothelial adhesion molecule expression is inhibited by CTRP3. (**A**) MyEnd (myocardial endothelial) cells were stimulated with LPS (50 ng/mL) for the indicated period. *E-selectin* and *P-selectin* mRNA levels were analyzed by real-time PCR. * *p* < 0.05 vs. con = unstimulated control, *n* = 4–6. (**B**) MyEnd cells were stimulated with LPS (lipopolysaccharides, 50 ng/mL) alone or pretreated with CTRP3 (C1q/TNF-related protein 3, 10 µg/mL, for 30 min) and with CTRP3 alone for 3 h. *E-selectin* and *P-selectin* mRNA levels were analyzed by real-time PCR. * *p* < 0.05 vs. con = unstimulated control, ^#^ *p* < 0.05 vs. LPS, *n* = 4–6.

Next, we investigated the effect of CTRP3 on major endothelial adhesion molecules which are crucial for the adhesion of leukocytes to the endothelium during infection and inflammation. Similar to cytokines, LPS induced the expression of vascular cell adhesion molecule-1 (*Vcam-1*) and intercellular adhesion molecule-1 (*Icam-1*) in MyEnd cells starting at 3 h after the start of stimulation (Figure 4A). CTRP3 alone had no effect on *Vcam-1* and *Icam-1* expression levels but significantly inhibited their LPS-induced expression (Figure 4B). Similarly, the release of the soluble forms of sVCAM-1 and sICAM-1 into the supernatant of MyEnd cells following LPS stimulation was abolished by CTRP3 (Figure 4C). In addition, we investigated the endothelial leukocyte adhesion molecules *E-selectin* and *P-selectin* in MyEnd cells. Both were similarly induced by LPS, *E-selectin* already after 1 h and *P-selectin* after 3 h of stimulation (Figure A2A), and their LPS-induced expression was abrogated by CTRP3 (Figure A2B). Subsequently, CTRP3 significantly inhibited the LPS-induced adhesion of monocytic J774.A1 cells to a monolayer of MyEnd cells (Figure 5).

### 3.4. Effects of CTRP3 In Vivo and on Tlr4 Expression in Endothelial Cells

**Figure 6:**
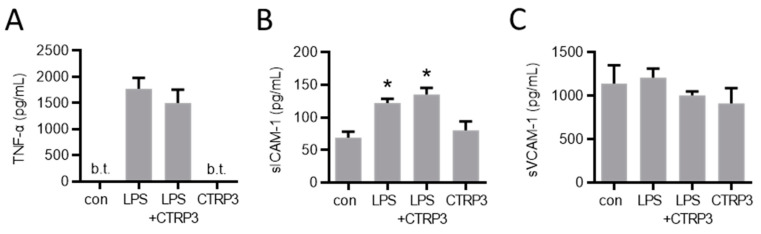
LPS-induced systemic markers of endothelial inflammation are not affected by CTRP3 in vivo. Male C57BL/6J wild-type mice were treated with i.p. injection of recombinant CTRP3 (C1q/TNF-related protein 3, 10 µg/animal) 30 min prior to i.p. LPS-injection (lipopolysaccharides, 1 µg/animal) for 2 h. LPS and CTRP3 alone were used as controls. (**A**) TNF-α (tumor necrosis factor-α), (**B**) sICAM-1 (soluble intercellular adhesion molecule-1) and (**C**) sVCAM-1 (soluble vascular cell adhesion molecule-1) protein levels in blood serum were quantified by ELISA. * *p* < 0.05 con = unstimulated control, *n* = 3–9.

In order to investigate the potential effects of CTRP3 in vivo, we injected a low dose of LPS (1 µg per animal) to induce a moderate systemic inflammatory response in male C57BL/6J mice. Circulating TNF-α and sICAM-1 levels were induced upon intraperitoneal LPS injection, whereas sVCAM-1 levels remained unaffected (Figure 6). The injection of recombinant CTRP3 (10 µg per animal) prior to LPS did not suppress the elevated serum levels of these circulating factors, indicating that CTRP3 was not capable of mitigating LPS-induced systemic inflammation.

**Figure A3:**
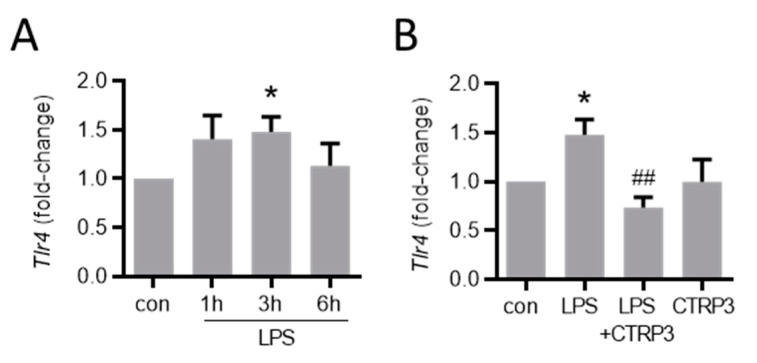
LPS-induced *Tlr4* expression is inhibited by CTRP3. (**A**) MyEnd (myocardial endothelial) cells were stimulated with LPS (lipopolysaccharides, 50 ng/mL) for the indicated period. *Tlr4* (Toll-like receptor 4) mRNA levels were analyzed by real-time PCR. * *p* < 0.05 vs. con = unstimulated control, *n* = 4–6. (**B**) MyEnd cells were stimulated with LPS (50 ng/mL) alone or pretreated with CTRP3 (C1q/TNF-related protein 3, 10 µg/mL, for 30 min) and CTRP3 alone for 3 h. *Tlr4* mRNA levels were analyzed by real-time PCR. * *p* < 0.05 vs. con = unstimulated control, ^##^ *p* < 0.01 vs. LPS, *n* = 4–6.

Finally, we investigated the potential mechanistic aspects for the inhibitory impact of CTRP3 on LPS-induced effects. To date, no specific receptor for CTRP3 has been identified and it remains unknown how CTRP3 precisely inhibits LPS-induced effects. In endothelial cells, LPS induced a slight yet significant increase in the mRNA expression of its receptor, *Tlr4* (Figure A3A). Notably, CTRP3 completely abolished this effect (Figure A3B), suggesting that the inhibition of *Tlr4* induction was involved in the LPS-antagonizing effects of CTRP3 in endothelial cells.

## 4. Discussion

The present study investigated the immune-regulatory functions of the pleiotropic adipokine CTRP3 from a perspective of cardiovascular biology, focusing on aspects of endothelial dysfunction. To provide the basis for functional studies, we performed a detailed analysis of *Ctrp3* expression in various tissues und cell types potentially involved in vascular inflammation. A tissue gene expression scan in wild-type mice revealed significant *Ctrp3* mRNA levels not only in classical white adipose tissues (gonadal and subcutaneous compartments), but in a number of very different tissues and organs, including the gastro-intestinal and urogenital tract, lung, thyroid gland, bone marrow, and the aorta. Most surprisingly, considerably higher *Ctrp3* gene expression levels were detected in the latter tissues when compared to white adipose tissue. These findings strongly argue against adipose tissue as the dominant site of *Ctrp3* expression (at least in mice) leaving the status of CTRP3 as a classical adipokine in question. These results and conclusions are in logical accordance with the recently published observation that adipocyte CTRP3 deficiency does not significantly alter systemic CTRP3 concentrations, indicating alternative and likely more potent sources for the circulating protein levels [14]. Importantly, the observation of pronounced *Ctrp3* expression in well-perfused tissues and organs, especially in the aorta, suggests a putative role of this protein in endothelium-related processes. Thus, we applied gene expression analysis in endothelial cells—including primary murine endothelial cells, murine MyEnd endothelial cells, and human endothelial cells (HUVECs)—and detected moderate *Ctrp3* mRNA levels under basal conditions. Given the known immune-regulatory properties of CTRP3 in adipose inflammation [20,22], we similarly hypothesized an involvement in endothelial inflammation. In cellular experimental settings which mimic endothelial inflammation following the bacterial infection in MyEnd cells in vitro, we most interestingly observed a strong and transient induction of *Ctrp3* expression by LPS stimulation after 1 h. In contrast, expression levels of the cytokines *Il-6* and *Tnf-α* stepwise increased after 3 and 6 h of LPS stimulation. We therefore concluded that LPS-induced, short-term *Ctrp3* expression preceding pro-inflammatory cytokine induction might represent an endogenous anti-inflammatory mechanism in endothelial cells. In order to verify this hypothesis, co-stimulation experiments were applied, incubating MyEnd cells with exogenous recombinant CTRP3 prior to LPS treatment. Of note, the results revealed the almost complete abrogation of LPS-induced *Il-6* and *Tnf-α* expression, thus indicating the potent anti-inflammatory properties of CTRP3 as an LPS antagonist in endothelial cells.

The expression of adhesion molecules in endothelial cells is crucial for the effective adhesion of leukocytes, such as blood monocytes, to the endothelium and subsequent infiltration to the site of infection [28,29]. Thus, *Icam-1*, *Vcam-1*, *E-selectin*, and *P-selectin* represent key factors in endothelial inflammation and monocyte recruitment [29,30]. We therefore studied the expression of adhesion molecules in endothelial cells upon LPS stimulation, observing a strong and persistent increase (~10–20 fold) for *Icam-1*, *Vcam-1*, and *E-selectin*. Most interestingly, LPS-induced *Icam-1* and *Vcam-1* mRNA levels, as well as the release of the soluble proteins, sICAM-1 and sVCAM-1, were significantly inhibited by co-treatment with exogenous CTRP3. Similarly, increased *E-selectin* and *P-selectin* expression levels were effectively attenuated in the presence of CTRP3. In order to investigate the functional impact of these molecular processes under pro-inflammatory conditions, a cellular co-culture model of endothelial inflammation was applied, assessing the monocyte adhesion to an endothelial cell layer. While LPS-treatment, as expected, substantially increased the rate of stable monocyte–endothelial cell adhesion, this effect was significantly inhibited in the presence of exogenous CTRP3, resulting in considerably reduced numbers of adherent monocytes (~50%). Overall, our data from in vitro experiments consistently indicated the potent anti-inflammatory properties of CTRP3 in LPS-induced endothelial inflammation on the levels of cytokine expression as well as monocyte adhesion. These intriguing findings establish CTRP3 as an endogenous antagonist of endothelial inflammation driven by bacterial infection and a potent immune-regulatory factor in the direct interaction of the inflamed endothelium with monocytes, similar to its well-characterized anti-inflammatory role in adipocyte-monocyte crosstalk in adipose inflammation [20,22].

Whereas the inhibitory effect of CTRP3 on LPS/TLR4-mediated inflammatory pathways has been described earlier [20], the exact mechanisms underlying this inhibition are still largely unknown. Endogenous CTRP3 might have an intracellular inhibitory effect on TLR4 signaling by downstream mechanisms. However, the results obtained by exogenous CTRP3, as in the present study, suggest an extracellular mode of molecular interaction. The recognition of LPS is a complex process and involves, in addition to the actual signal receptor TLR4, the soluble LPS-binding protein (LBP) and the phospholipid anchored membrane protein CD14 [31]. Although this was not the focus of the present study, our data suggest that the inhibition of LPS-induced *Tlr4* upregulation might be involved in the LPS-antagonizing effect of CTRP3 in endothelial cells, thereby preventing a potentiation of LPS-induced inflammatory signaling.

We further aimed to investigate the situation under pathophysiological conditions in vivo and applied a murine model with a moderate systemic inflammatory response syndrome (SIRS) induced by the intraperitoneal injection of LPS. We already observed the inhibitory effects on IL-6 and macrophage inflammatory protein-2 (MIP-2) levels in a similar experimental setting with the additional administration of exogenous CTRP3 prior to LPS [21]. Therefore, we assessed the regulation of circulating proteins with respect to signs of endothelial inflammation. While the systemic concentrations of the pro-inflammatory cytokine TNF-α and the soluble adhesion molecule sICAM-1 strongly increased upon LPS stimulation, no effects of CTRP3 were observed. These results are somewhat surprising since we observed clear effects in endothelial cells in vitro in the present study and, though less pronounced, effects on circulating IL-6 and MIP-2 levels in vivo [21]. This might indicate that either the administered dose of CTRP3 (10 µg/animal) was insufficient or the site/mode of application (intraperitoneal injection) was inopportune to obtain significant inhibitory effects on LPS-induced circulating levels of markers of endothelial inflammation.

We are aware that our study may have further limitations. We used recombinant human CTRP3 to block LPS-mediated effects in murine cells, yet did not consider this as a major problem since the homology of amino acid sequences is 97.7% and the inhibitory effect would likely be rather stronger with murine CTRP3. Moreover, cell culture experiments using only one cell-type and applying pre-incubation with the inhibitor cannot precisely reflect the in vivo situation. Ultimately, we can solely speculate on LPS-mediated effects but not on alternative TLR4 ligands, or even other TLR ligands. Furthermore, future approaches elaborating on the present data should also address the effects of LPS on systemic CTRP3 levels.

The vascular endothelium plays an important role in the organization of the immune response. In sepsis, the endothelium is involved in cytokine overproduction, leading to a cytokine storm and endothelial dysfunction [32]. Thus, from a clinical perspective, an anti-inflammatory therapy with CTRP3 in endotoxemia/sepsis is conceivable. Moreover, TLRs are not only activated by pathogen-associated molecular patterns (PAMPs) such as LPS but also by damage-associated molecular patterns (DAMPs) as they occur during tissue damage [33], for example vascular tissue damage following minimal interventional angioplasty, as it is frequently performed in cardiovascular patients. Thus, in perspective, CTRP3 could potentially be used to limit the vascular inflammatory process after angioplasty, e.g., on a drug-eluting stent.

## 5. Conclusions

The present study characterizes CTRP3, beyond its classical function as an immune-regulatory adipokine, as a protein widely expressed in various tissues and involved in vascular inflammation. CTRP3 expression in endothelial cells is swiftly increased by inflammatory stimulation and the protein exerts anti-inflammatory functions as an inhibitor of LPS-induced endothelial inflammation. The endothelial expression of adhesion molecules and pro-inflammatory cytokines, as well as monocytic cell adhesion on inflamed endothelial cell layers, is significantly inhibited in the presence of CTRP3 (as is summarized in the graphical abstract). Taken together, our present data strongly suggest that CTRP3 represents a potent immune-regulatory factor in endotoxemia-induced vascular inflammation.

## Data Availability

The data presented in this study are available on request from the corresponding author.
